# Analysis of the influence of passenger load on bus energy consumption a vehicle-engine combined model-based simulation framework

**DOI:** 10.1038/s41598-022-18866-6

**Published:** 2022-08-25

**Authors:** Xinfeng Yang, Lanfen Liu

**Affiliations:** School of Traffic and Transportation Engineering, Lanzhou Jiaotong University, Lanzhou, 730070 People’s Republic of China

**Keywords:** Environmental sciences, Energy science and technology

## Abstract

Analyzing of the energy consumption (EC) in bus operation is important for reducing operating costs, improving sustainable solutions and creating environmentally friendly cities. The purpose of this paper is to identify the factors, such as passenger load, speed and acceleration, that affect significantly EC in bus operation. This paper builds a simulation framework for describing the level of energy based on the vehicle-engine combined (VEC) EC model. On the basis of the relationship between engine torque, power, traction and EC, the simulation framework mainly includes the road model, vehicle model, engine model and driver strategy. Furthermore, the correlations between energy consumption, passenger load, vehicle speed and acceleration are analyzed in different station spacing. The results show that the passenger load has a significant impact on EC of buses, and is related to the vehicle’s speed and acceleration. Generally, the higher the maximum driving speed, the higher the EC of the bus. Acceleration strategies and maximum speed limits are critical factors determining the EC of bus for a certain passenger load and station spacing. For the same station spacing and maximum driving speed, the acceleration phase is under a greater contribution to the increase of EC. In addition, the greater the maximum speed limit or the acceleration, the greater the contribution percentage of EC increase in the acceleration phase. The simulation framework based on vehicle-engine combined EC model and specific fuel consumption maps can obtain the operating EC of buses for situations with different station spacing and maximum speed, which is conducive to vehicle operation EC analysis. Acceleration strategies and maximum speed limits are critical factors determining the EC of bus for a certain passenger load and station spacing. Therefore, energy savings can be obtained by optimizing the driving strategy.

## Introduction

The rapid development of motor vehicles will affect the energy consumption of urban transportation, leading to serious environmental problems^1^. The sectoral share of total final consumption has remained relatively stable compared to recent years. The transportation sector on EC accounts for about 28% of all world EC, second only to that of industry^2^. Fuel consumption of the global transportation will continue to increase, and the daily consumption will reach 60 million barrels accounting for 61% of the total output of fuel oil in 2035^3^. The transport sector in the United States accounts for 70% of its petroleum consumption^4^. In 2015, the average emission of passenger cars in Europe was slightly higher than 120 g CO_2_ / km, which means an additional 36 ~ 48 g CO_2_/ km, or an increase in fuel consumption of about 1.5 ~ 2L / 100 km (petrol equivalent)^5^. The U.S. of Transportation Statistics reported that between 1960 and 2012, bus fuel consumption continued to increase from 827 million gallons/year to 2059 million gallons/year^6^. The share of EU buses in all the heavy vehicles is 11%, accounting for 15% of the total fuel consumption of all the heavy vehicles^7^.

For bus operation companies, fuel is an important aspect of their total cost. Optimizing vehicle utilization is one of the main concerns of bus operators in order to reduce fuel consumption^8^. Effective policies to promote the efficient use of energy by reducing fuel consumption or reducing the generation of greenhouse gases are the foundation for achieving sustainability goals. A large number of studies have confirmed that the fuel consumption of buses depends on many factors, such as road type, road slope, air conditioning, speed, acceleration, load mass, driving style and so on, among which speed and load mass are the most important factors^9–13^. Therefore, identifying factors such as passenger load, speed and acceleration has a significant impact on EC in bus operation, which is a meaningful (and potentially powerful) management tool for these companies. Moreover, it can find out the way to reduce the EC, and provide scientific theoretical support for selecting dispatching strategy and improving the energy efficiency of bus.

In the aspect of EC analysis for bus, some scholars have studied and made some achievements. Modeling of EC for transit buses is a very important issue. There are three major types of EC prediction models: the regression model, VSP-based EC model (Vehicle Specific Power) and the mechanical model.

*The regression model*: This kind of model establishes the relationship between operational conditions and EC, through different regression methods and alternative parameters. For example, Ahn et al.^14^ estimated vehicle fuel consumption and emissions based on instantaneous speed and acceleration levels by using a hybrid regression prediction model. Cai et al.^15^ established a fuel consumption calculating model for passenger vehicles under the basic conditions considering the influence of mass and speed. Peng et al.^16^ developed an urban transport model for energy consumption and emission based on LEAP (long range energy alternatives planning system). Tang et al.^17^ established an energy consumption model for urban bus transport by introducing the energy cost rate parameters considering the life cycle of the public vehicle energy consumption. Okafor I F et al.^18^ calculated the energy efficiency of the sample vehicles with gasoline and diesel engines for public passenger transport in Nigeria, and developed models to estimate the energy efficiency of the sample vehicles using statistical regression technology on SPSS. Masikos et al.^19^ designed GRNN (General Regression Neural Networks) for approximating the specificities between the factors identified as major contributors in vehicular consumption, and proposed a vehicular consumption prediction model. Ivkovic et al.^20^ calculated the fuel consumption and fuel costs for these three different bus technologies of the Republic of Serbia by selecting correction factors for fuel consumption.

This kind of model mainly reflects the EC level of transit buses through selecting correction factors (operating distance or average speed) of fuel consumption for the whole region. However, it is not sensitive to the operation conditions and passenger load of a single vehicle, so it is difficult to apply it to an analysis of the impact of passenger load, speed and acceleration on bus operation EC.

*VSP-based EC model*: This kind of model takes into account the changes of kinetic energy, potential energy, rolling friction resistance and air resistance, and obtains the vehicle operation EC through collecting the relationship between the VSP and EC of vehicles. There are lots of valuable researches in this kind of model. Song et al.^21^ proposed a mathematical model for developing the VSP distributions based on the average travel speed by using large samples of floating car data collected from the expressways in Beijing. This model can be integrated efficiently with traffic models or data for estimation of fuel consumption and emissions. Frey et al.^9^ used a VSP-based approach for modeling fuel consumption of diesel and hydrogen buses and VSP-based modal average fuel consumption rates are compared. Wu et al.^22^ designed a method to estimate fuel consumption by integrating VSP and CAN bus technology because of the accessibility and stability of the controller area network bus. Zhang et al.^11^ estimated the fuel consumption and CO_2_ emission factors under a typical bus driving cycle in Beijing by use of an operating mode binning methodology which was defined by VSP and vehicle speed. Holmén, Sentoff^23^ compared tailpipe CO_2_ emissions and fuel consumption of an HEV (Hybrid-electric vehicle) passenger car to a conventional vehicle by using VSP assignments. For more about the VSP-based EC model, the reader can refer to those of Wang et al.^10^, Duarte et al.^24^, Wang and Rakha^25^, Xu et al.^26^ and Huan et al^1^.

The VSP method integrates the effects of road slope, speed and acceleration into one parameter, which can explain the main changes in fuel consumption. The VSP-based model is helpful to describe bus trips and their fuel consumption^9^. Therefore, it has been widely concerned and applied in the estimation of EC and emission. Many works using the VSP method take into account a variable number of passengers, e.g. Yu et al.^27^ quantified the influence of passenger load on diesel bus emissions and fuel consumption based on VSP based modeling approach. Chen et al.^28^ analysed the influence of traffic congestion and passenger load on feeder bus fuel and emissions. Fredy et al.^29^ studied the effects of passenger load, road grade, and congestion level on the fuel consumption and emissions from a Euro VI compressed natural gas (CNG) urban bus and a Euro V diesel urban bus.

*Vehicle-engine combined EC model*: Based on engine universal characteristic, vehicle-engine combined model mainly considers the characteristics of the road, vehicle, engine and an operating mode, and can obtain the micro EC of the vehicle under different road conditions and passenger capacity^30^. Such as, He et al.^31^ set up a mathematical model of engine universal characteristics to analyze the influence degrees of driving speed, loading mass and road resistance coefficient on automobile fuel economy. Bao^32^ built the engine mathematical model based on the evaluation method of vehicle fuel economy, and established a calculation process and a method of constant speed fuel consumption based on the graphical method of fuel consumption curve. Chaim, Shmerling^33^ proposed a mathematical model for estimating the fuel consumption of vehicles of 100 km under standard operating conditions, and calculated the fuel consumption of vehicles in average speed, acceleration, deceleration and idle mode respectively. Ma et al.^12^ established a combined fuel consumption model based on driving characteristics and automobile engine based on vehicle-engine combined model after analyzing the influence of driving mode on fuel consumption. Ehsani et al.^30^ established a mechanical model considering the conditions of temperature, driving mode and road type, and analyzed the fuel consumption and CO_2_ emission of different types of vehicles. Schwickart et al.^34^ proposed an energy consumption model of electric vehicles, based on vehicle longitudinal dynamics and its energy consumption. Yang et al.^35^ proposed a mechanical model for describing the energy levels used by different vehicles based on the universal characteristics of the engine, which takes into account the characteristics of the road, vehicle, engine and driving mode.

Vehicle-engine combined (VEC) model can obtain the relationship between the engine instantaneous state and EC based on engine universal characteristic considering the characteristics of engine, road and vehicle, and can track the change of vehicle's instantaneous EC, which is conducive to vehicle operation EC analysis. For public transit buses, the passenger load should not be ignored for estimating bus energy consumption because the load changes during the journey^24^, and it is not possible to characterize all vehicles in actual operation. The VEC model can obtain the micro energy consumption of vehicles under different road conditions and passenger load. So, we used the VEC model in this paper to describe the level of energy used in different vehicles.

In this study, a simulation framework for describing the level of energy used in different vehicles is provided based on the VEC model, and this framework mainly includes the road model, vehicle model, driver strategy and engine model. Furthermore, the correlations between EC, passenger load, vehicle speed and acceleration are evaluated by using this simulation framework. Some comparisons are made for the EC correlations among maximum speed, station spacing and passenger load. VEC model can effectively reflect the driver's operation strategy comparison VSP-based EC model, and the impact of different driving conditions on energy consumption can be analyzed quantitatively, which is helpful to find out some factors to reduce bus energy consumption. In addition, the simulation framework is easy to be combined with the dispatching model, which can provide support for the research of bus energy-saving dispatching.

### The simulation framework for EC analysis

**Figure 1:**
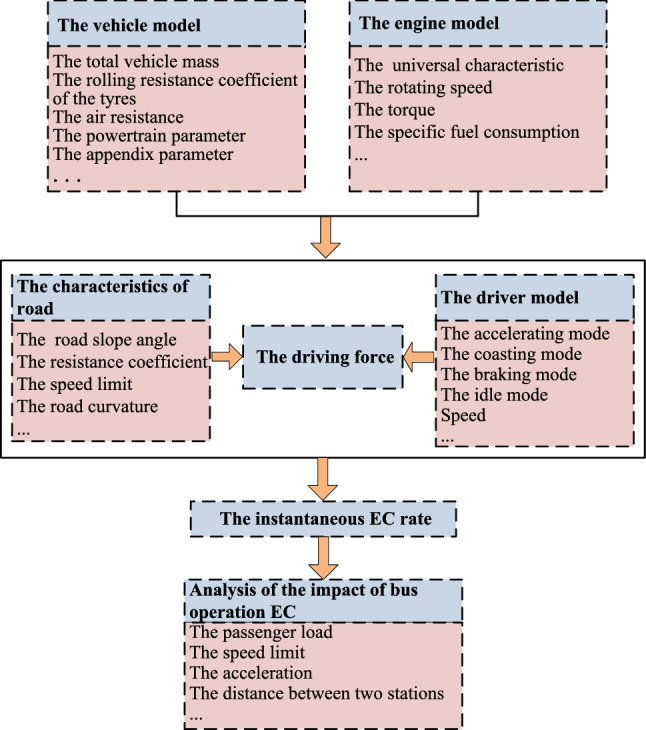
The simulation framework based on VEC model.

The simulation framework we used is built on vehicle-engine combined EC model. On the basis of the relationship between engine torque, power, traction and EC, the simulation framework mainly includes the vehicle model, engine model and driver strategy. The actual driving state of the transit bus is represented by the instantaneous driving condition, such as driving speed, acceleration, etc., and the passenger load. Given this information, the operating EC of transit bus can be obtained. The structure of the simulation framework based on the VEC model is depicted in Fig. 1.

### Notations

Notation for the following discussion is given in Table 1.

**Table 1:** Main parameters.

Parameters	Description	Parameters	Description
$F_{t}$	The driving force of the vehicle (N)	$T_{e}$	The torque (N·m)
$F_{r}$	The total rolling resistance of all the wheels (N)	$n_{e}$	The engine rotating speed (r/min)
$F_{w}$	The air resistance (N)	$P_{e}$	The propulsion power required by the driving wheels (kW)
$F_{g}$	The grade resistance (N)	$\frac{{{\text{d}}v}}{{{\text{d}}t}}$	The acceleration of vehicle, (m/s^2^)
$F_{j}$	The acceleration resistance (N)	$\eta_{t}$	The mechanical efficiency
$F_{k}$	The additional rolling resistance (N)		
$\alpha$	The road slope angle		
*m*	The total mass of the vehicle (kg)		
*f*	The rolling resistance coefficient of the tires		
$L$	The wheelbase of the vehicle (mm)	$Q_{s}$	The EC rate of a vehicle (g/sec)
$a$	The distance of the mass center from the front axle (mm)	$g_{e}$	The specific EC (g/(kW·h))
*A*	The frontal area of the bus (m^2^)	$\phi$	The road adhesion coefficient
$C_{D}$	The drag coefficient of the air	$T_{e}$	The torque (N·m)
*v*	The driving speed (km/h)	$n_{e}$	The engine rotating speed (r/min)
$i_{gi}$	The speed ratio of gearbox for each gear, ($i = 1,2...p,$ *p* is the number of gears)	$P_{e}$	The propulsion power required by the driving wheels (kW)
$i_{0}$	The driving axle	$F_{t\cdot highest}$	The driving force in the highest gear (N)
*r*	The dynamic rolling radius (m)	$F_{n}$	The normal reaction force of the driving wheels (N)

### The vehicle model

**Figure 2:**
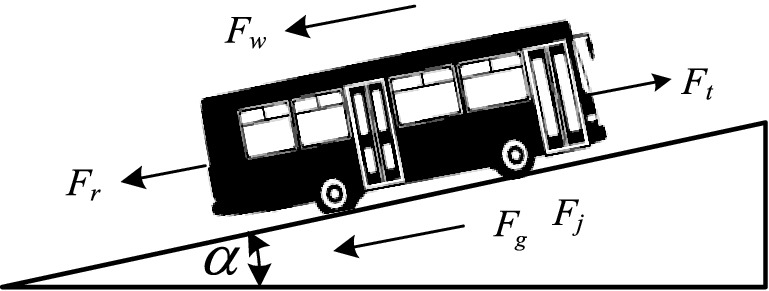
The forces that affect the vehicle during motion.

The movement of a bus is determined by various external forces acting on the vehicle. Figure 2 shows the forces that affect the vehicle during motion.

The main forces acting on the vehicle in the longitudinal direction are the tractive force and the driving resistance forces.

According to the balance relationship of these forces, the bus driving forces equation can be established as follows:^34,36^

1 $$ F_{t} = F_{r} + F_{k} + F_{g} + F_{w} + F_{j} \quad ({\text{N}}). $$

#### (1) The road resistance

Road resistance includes the rolling resistance and the grade resistance. The rolling resistance force $F_{r}$ depends on the force that presses the vehicle onto the surface and depends on the road slope angle. For roads with a slope of less than 30%, equivalent to a slope angle less than $17^{ \circ }$, the road resistance force can be simplified as follows:

2 $$ F_{r} + F_{g} = mgf + mg\sin \alpha \quad ({\text{N}}), $$

where *g* is the gravitational constant (N/kg). The total mass of the vehicle is the sum of vehicle curb weight $m_{c}$ and passenger load $m_{p}$.

3 $$ m = m_{c} + m_{p} = m_{c} + n \cdot w_{p} \quad ({\text{N}}), $$

where *n* is the number of passengers on the vehicle and $w_{p}$ is the average passenger weight (kg).

When driving on a curve, there will be an additional rolling resistance $F_{k}$ which can be computed as:

4 $$ F_{k} = m\frac{{v^{2} }}{R}\left( {\frac{L - a}{L}\sin \beta_{f} + \frac{a}{L}\sin \beta_{r} } \right)\quad ({\text{N}}), $$

where *R* is turning radius (m), $\beta_{f}$,$\beta_{r}$ represents the sideslip angle of front and rear tires, respectively.

#### (2) The air resistance

The air resistance force $F_{w}$ is the component of the air force in the driving direction when the bus is running, which is related to the shape of the projected frontal surface of vehicle, the drag coefficient of the air and the air density^34,36^.

5 $$ F_{w} = \frac{{C_{D} Av^{2} }}{21.15}\quad ({\text{N}}). $$

#### (3) The acceleration resistance

When the vehicle accelerates, the inertial mass forces appear in the opposite direction of the acceleration. The acceleration of the vehicle in longitudinal direction can be computed as:

6 $$ F_{j} = \delta_{i} m\frac{{{\text{d}}v}}{{{\text{d}}t}}\quad ({\text{N}}), $$

where $\delta_{i}$ is the correction coefficient of rotating mass ($\delta_{i} > 1$), which is mainly related to the moment of inertia of flywheel and wheel, and the transmission ratio.

The power requirement for a given vehicle can be calculated from the above road resistance equations, and Eq. 12 shows the road load equation:

7 $$ P_{e} = \frac{v}{{3600\eta_{t} }}\left( {mgf + mg\sin \alpha + \frac{{C_{D} Av^{2} }}{21.15} + \delta m\frac{{{\text{d}}v}}{{{\text{d}}t}}} \right) = \frac{1}{{\eta_{t} }}\left( {\frac{mgfv}{{3600}} + \frac{mg\sin \alpha v}{{3600}} + \frac{{C_{D} Av^{3} }}{76140} + \frac{\delta mv}{{3600}}\frac{{{\text{d}}v}}{{{\text{d}}t}}} \right)\quad ({\text{kW}}). $$

### The engine model

The universal characteristic of the engine is the basic data for the simulation calculation of vehicles EC. Furthermore, the specific EC can be determined according to rotation speed and torque based on the universal characteristic of the engine.

The performance maps of universal characteristics of engine are always given in the form of a family of curves, which can show the variation relationship of the main parameters (the specific fuel consumption, rotation speed and torque) of the engine over the full range of its operating conditions. Therefore, when determining the specific fuel consumption, it can be obtained by interpolation method according to the rotation speed and torque in a certain working state of the engine from the performance maps of universal characteristics. In addition, the rotation speed can be calculated from the relationship of vehicle speed and engine rotating speed^32^.

8 $$ n_{e} = \frac{{vi_{gi} i_{0} }}{0.377r}\quad ({\text{r/min}}), $$

Furthermore, the torque $T_{e}$ can be calculated by

9 $$ T_{e} = \frac{{9550P_{e} }}{{n_{e} }}\quad ({\text{N}} \cdot {\text{m}}), $$

So, the specific EC $g_{e}$ can be determined according to the engine rotating speed $n_{e}$ and torque $T_{e}$, and the EC rate of a vehicle can be calculated by

12 $$ Q_{s} = \frac{{P_{e} g_{e} }}{3600}\quad ({\text{g/sec}}). $$

So, we can find that the EC of a vehicle depends on the vehicle speed and the corresponding driving strategy under this speed. The driving strategy includes the selection of acceleration, gear selected and maximum driving speed, etc.

### Driving strategy

Wang et al.^37^ and Mahesh et al.^38^ found that city size, local road infrastructure, and driving behavior lead to significant differences in vehicle driving patterns among the cities. The driving behavior has a great effect on both emissions and fuel consumption^39^. In order to simplify the calculation, the driver is set to accelerate the vehicle at a certain acceleration. When the speed reaches the specified speed, the driver uses the uniform speed driving, and then the equal deceleration braking strategy is adopted in the process of entering the station. Gear-shifting speed of 1800 r/min was used in this study.

When the driving force of the vehicle is balanced with the driving resistance, the speed reaches the maximum. According to the balance equation of the driving force and driving resistance in the highest gear,

13 $$ F_{t \cdot highest} (v)\; = \;F_{r} (v)\; + F_{k} (v)\; + F_{w} (v)\; + F_{g} (v), $$

the maximum speed can be obtained by selecting the maximum value of the feasible speed.

However, the maximum speed is also limited by the maximum road adhesion. The normal reaction force of the driving wheels is calculated according to the following driving modes:

14 $$ F_{n} \; = \left\{ {\begin{array}{*{20}l} {mg,} \hfill & {\text{All - wheel drive,}} \hfill \\ {\frac{mg}{L}(L - a),} \hfill & {\text{Front - wheel drive,}} \hfill \\ {\frac{{G_{a} }}{L}a,} \hfill & {{\text{Rear - wheel drive}}{.}} \hfill \\ \end{array} } \right. $$

If

15 $$ \frac{{F_{t \cdot highest} }}{{F_{n} }} > \phi , $$

it means that the normal reaction force cannot meet the requirements of the calculated maximum speed. Thus, the maximum speed should be recalculated according to the normal reaction force $F_{n}$. Here, the road adhesion coefficient is generally 0.7–0.8 for asphalt pavement^40^.

### The characteristics of road

Generally, the average station spacing should be 500 ~ 800 m in urban area and 800 ~ 1000 m in a suburban area in China. In the downtown area, the passenger flow is relatively dense, so the station spacing should be smaller. However, in the urban fringe and suburbs, the population distribution is relatively scattered, and the station spacing can be appropriately increased^41^. In addition, some cities stipulate that the speed of buses in the city should not exceed 30 km/h, and the speed of BRT with special lanes should not exceed 60 km/h. Wang et al.^37^ analyzed driving characteristics for eleven cities in China, and found that the range of average speed during off-peak period was between 23 and 41 km/h; whereas during peak-hour the range of average speed was between 16 and 31 km/h. Therefore, the average station spacing is in the range of 400 ~ 1500 m and the maximum speed during the trip is set in the range of 30 ~ 60 km/h to analyze the EC of bus operation.

Three acceleration strategies are adopted when starting, which are $0.4\;{\text{m/s}}^{{2}}$, $0.6\;{\text{m/s}}^{{2}}$ and $0.8\;{\text{m/s}}^{{2}}$ respectively. The equal deceleration braking strategy of $- \;0.8\;{\text{m/s}}^{{2}}$ is adopted in the process of entering the station.

## The analysis of operation EC for Bus

**Figure 3:**
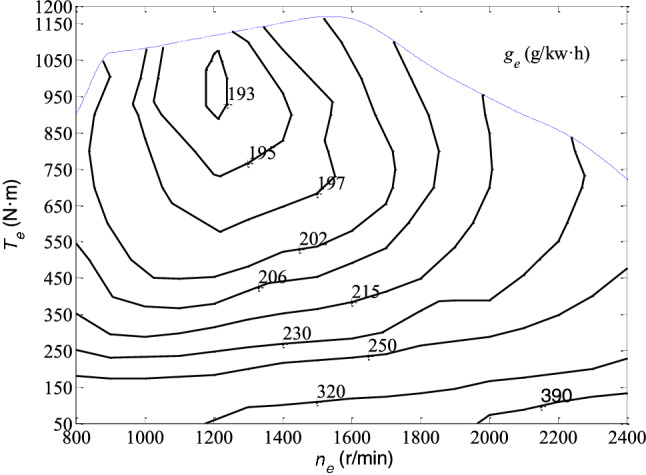
The performance map for WP7.270.

In this paper the Fonton AUV BJ6123C7MJB with engine WP7.270 is selected for specific analysis of EC by using the simulation framework based on VEC mode. The performance maps of universal characteristics for engine WP7.270 are shown in Fig. 3 and the main parameters of the test vehicle are listed in Table 2.

**Table 2:** Main parameters of the test vehicle.

Description	Value
curb weight (kg)	11,200
Gross vehicle weight (Kg)	17,600
Number of seats(persons)	37
Rated passenger capacity s(persons)	98
Front surface area *A* (m^2^)	7.78
Overall dimensions(*L* × *W* × *H*)(mm)	11,980 × 2550 × 3050
Tyre size	275/70R22.5
$i_{g}$	5.82, 3.23, 1.96, 1.26, 1.00
$i_{0}$	5.571
$\eta_{t}$	0.9
wheelbase of the vehicle (mm)	5900
distance of the mass center from the front axle (mm)	Full load:3848
	Empty: 4143

The feasible speed range of each gear with engine speed range from 800 to 2400 rpm can be obtained as showed in Table 3. In practice, the operation of public transport buses is complex, and the distance and speed limit of different bus lines are also different.

**Table 3:** The feasible speed range of each gear.

Gear	$i_{g}$	Speed range of Engine (rpm)	fEasible speed range (Km/h)
1	5.82	800–2400	4.33–12.98
2	3.23	800–2400	7.79–23.38
3	1.96	800–2400	12.84–38.53
4	1.26	800–2400	19.98–59.94
5	1	800–2400	25.17–75.52

Fuel consumption is most intuitively measured as the fuel required to travel a unit of distance (L/100 km). Fuel consumption per 100 km can of a bus can be obtained according to the instantaneous speed, the power requirement for a given bus and the EC rate in the performance maps of universal characteristics for an engine. So, the fuel consumption per 100 km of a bus can be calculated by

22 $$ G_{s} = l\sum\limits_{t} {\frac{{P_{e}^{t} g_{e}^{t} t}}{36\rho }} , $$

where $G_{s}$ represents the fuel consumption per 100 km (L/100 km), *l* is the distance of bus simulation (m), *t* is the unit simulation time, $P_{e}^{t}$ is the power at time *t*, $g_{e}^{t}$ represents the specific EC (g/(kW·h)) at time *t* and $\rho$ is the fuel density (kg / L). Here,$\rho$ = 0.835 kg/L.

### The EC of empty buses

**Figure 4:**
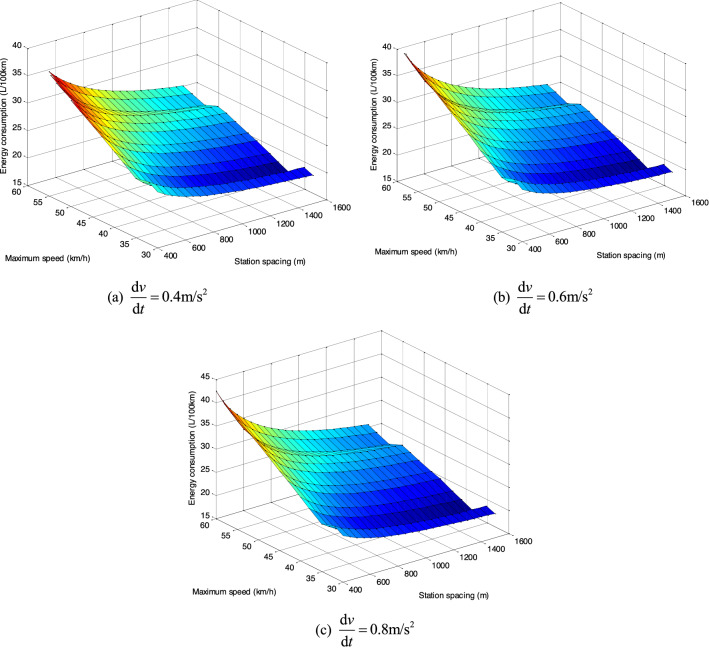
The EC of empty buses with different station spacings and maximum speeds.

Based on the simulation framework we designed based on vehicle-engine combined EC model and specific fuel consumption maps, the EC of empty buses can be obtained for situations with different station spacing and maximum speed through second by second simulation, which are shown in Fig. 4.

**Figure 5:**
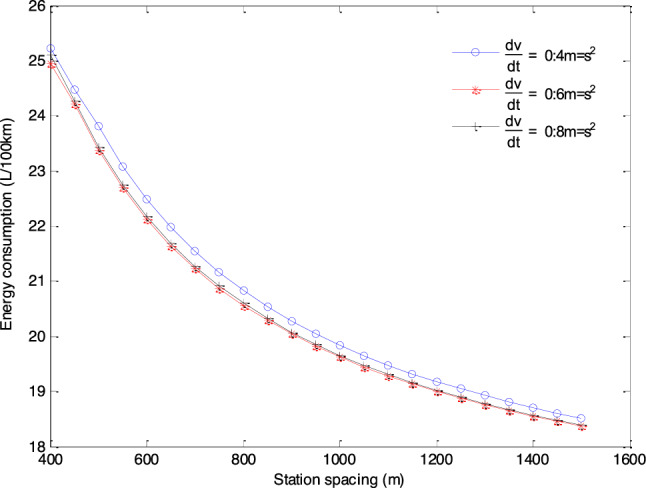
The lowest EC with different station spacings under different acceleration strategies.

The results presented in Fig. 4 show that the larger the average station spacing, the lower the EC per 100 km. Moreover, when the vehicle acceleration is $0.4\;{\text{m/s}}^{{2}}$ and $0.6\;{\text{m/s}}^{{2}}$, and the station spacing is 400 m, the maximum speed with the lowest EC per 100 km is 30 km/h. In other cases, the maximum speed with the lowest EC per 100 km is 35 km/h. Generally, the higher the maximum driving speed, the higher the EC of the bus. Under the three acceleration strategies, the comparison of the lowest EC per 100 km with different station spacings is shown in Fig. 5. As can be seen from Fig. 5, the lowest EC per 100 km with different station spacings is the acceleration strategy of $0.6\;{\text{m/s}}^{{2}}$.

**Figure 6:**
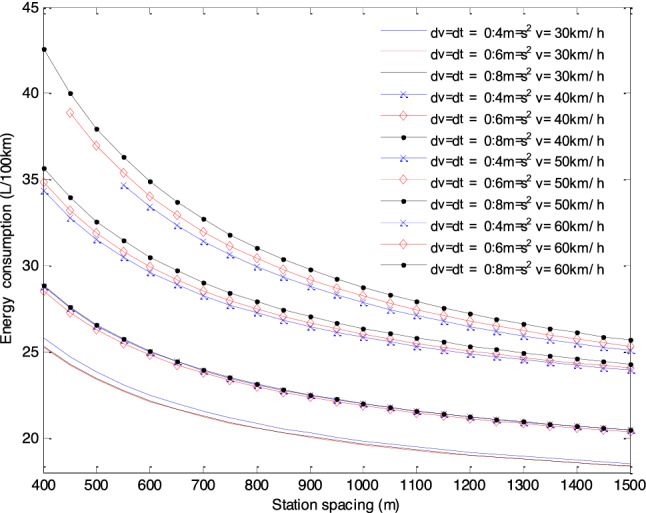
Contrastive analysis of EC under different acceleration strategies, station spacings and maximum speeds.

The EC of empty buses under different acceleration strategies, station spacing and maximum speed is shown in Fig. 6. For the lower maximum driving speed (*v* = 30 ~ 45 km/h), the lower EC of 100 km is the acceleration strategy of $0.6\;{\text{m/s}}^{{2}}$, but there is little difference among the three acceleration strategies. For the higher driving speed (*v* = 45 ~ 60 km/h), the difference of the EC of the three starting strategies increases gradually. In this case, the lower EC of 100 km is the acceleration strategy of $0.4\;{\text{m/s}}^{{2}}$, and the higher EC is the acceleration strategy of $0.8\;{\text{m/s}}^{{2}}$.

In addition, the smaller the average station spacing, the greater the difference of EC of 100 km. It can be seen that a smooth acceleration strategy is a more energy-saving driving strategy.

The EC of empty buses under different acceleration strategies in the acceleration phase is shown in Table 4. The results presented in Table 4 show that the higher the acceleration, the less EC to reach the maximum speed and the smaller acceleration distance in the acceleration phase.

**Table 4:** Analysis of EC in acceleration phase.

*v* (km/h)	EC (g)			Acceleration distance (m)		
	${\text{d}}v/{\text{d}}t = 0.4\;{\text{m/s}}^{{2}}$	${\text{d}}v/{\text{d}}t = 0.6\;{\text{m/s}}^{{2}}$	${\text{d}}v/{\text{d}}t = 0.8\;{\text{m/s}}^{{2}}$	${\text{d}}v/{\text{d}}t = 0.4\;{\text{m/s}}^{{2}}$	${\text{d}}v/{\text{d}}t = 0.6\;{\text{m/s}}^{{2}}$	${\text{d}}v/{\text{d}}t = 0.8\;{\text{m/s}}^{{2}}$
30	41.471	36.069	34.366	86.806	57.87	43.403
35	55.26	48.217	45.854	118.152	78.768	59.076
40	69.871	61.486	58.607	154.321	102.881	77.16
45	86.778	76.769	73.289	195.313	130.208	97.656
50	106.104	94.281	90.203	241.127	160.751	120.563
55	128.159	114.123	109.665	291.763	194.509	146.375
60	151.886	135.945	132.156	347.222	231.481	188.161

### The EC of buses with passenger load

**Figure 7:**
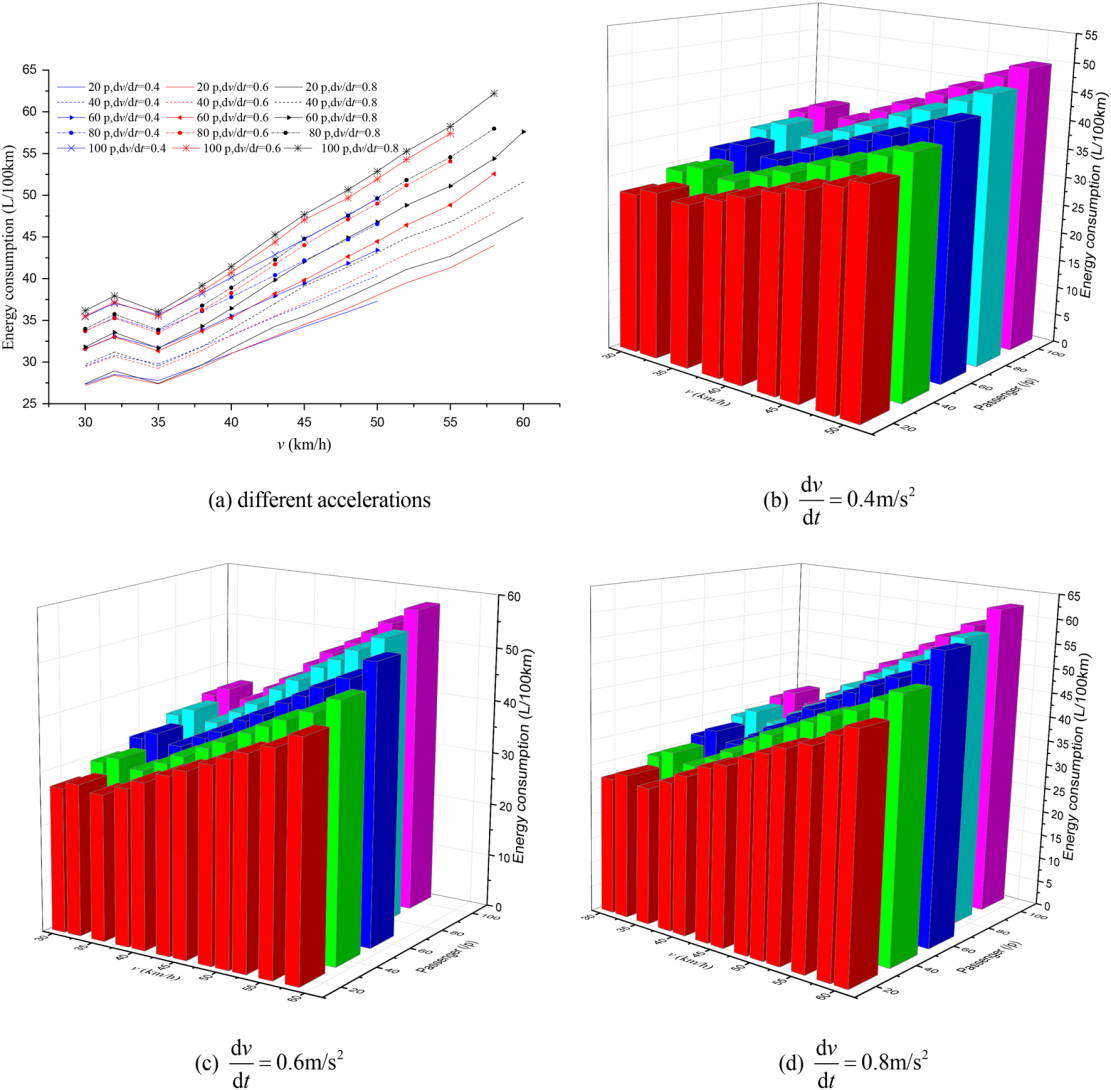
The EC of different passenger load, acceleration and maximum speed for 400 m station spacing.

**Figure 8:**
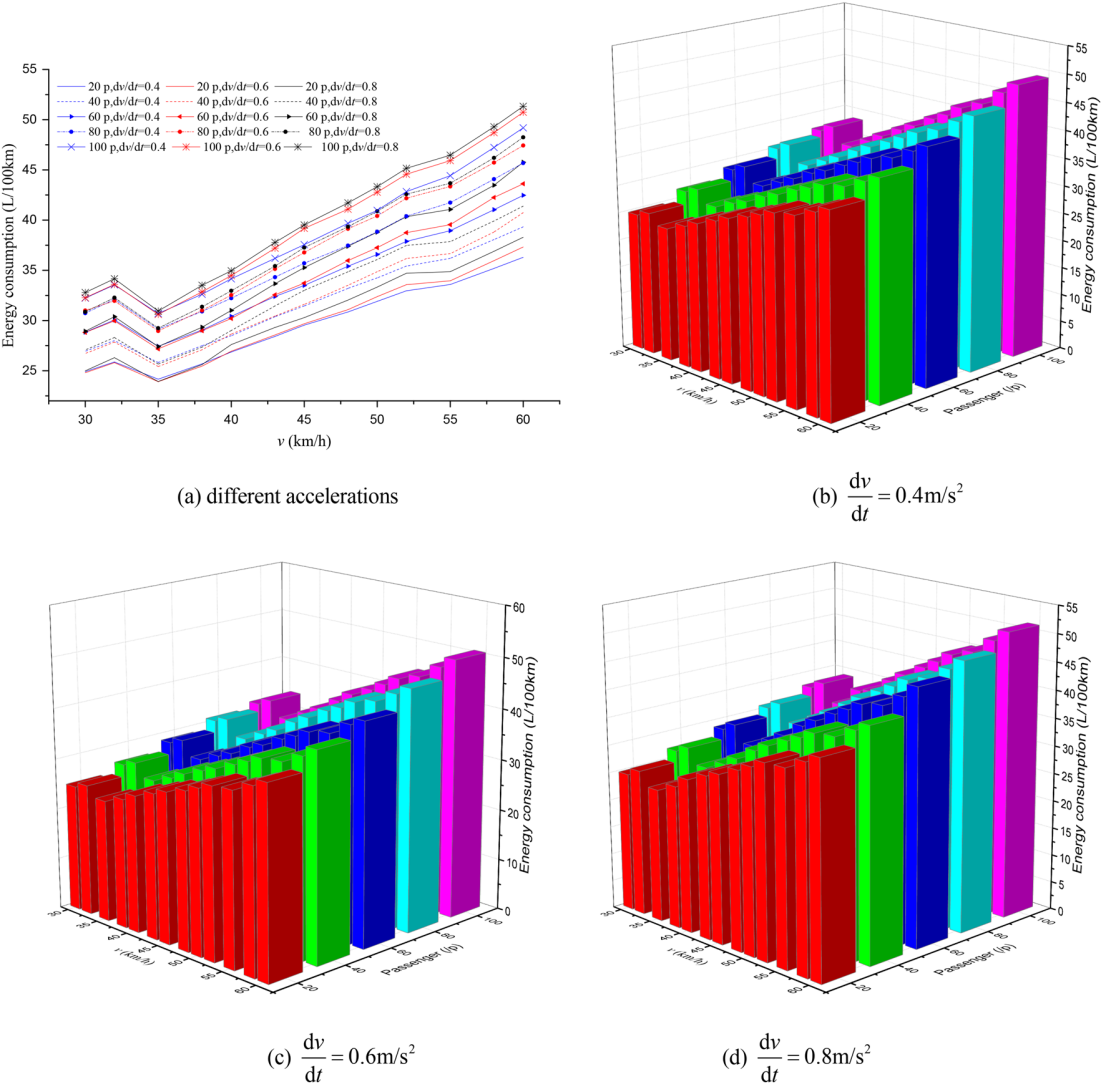
The EC of different passenger load, acceleration and maximum speed for 600 m station spacing.

**Figure 9:**
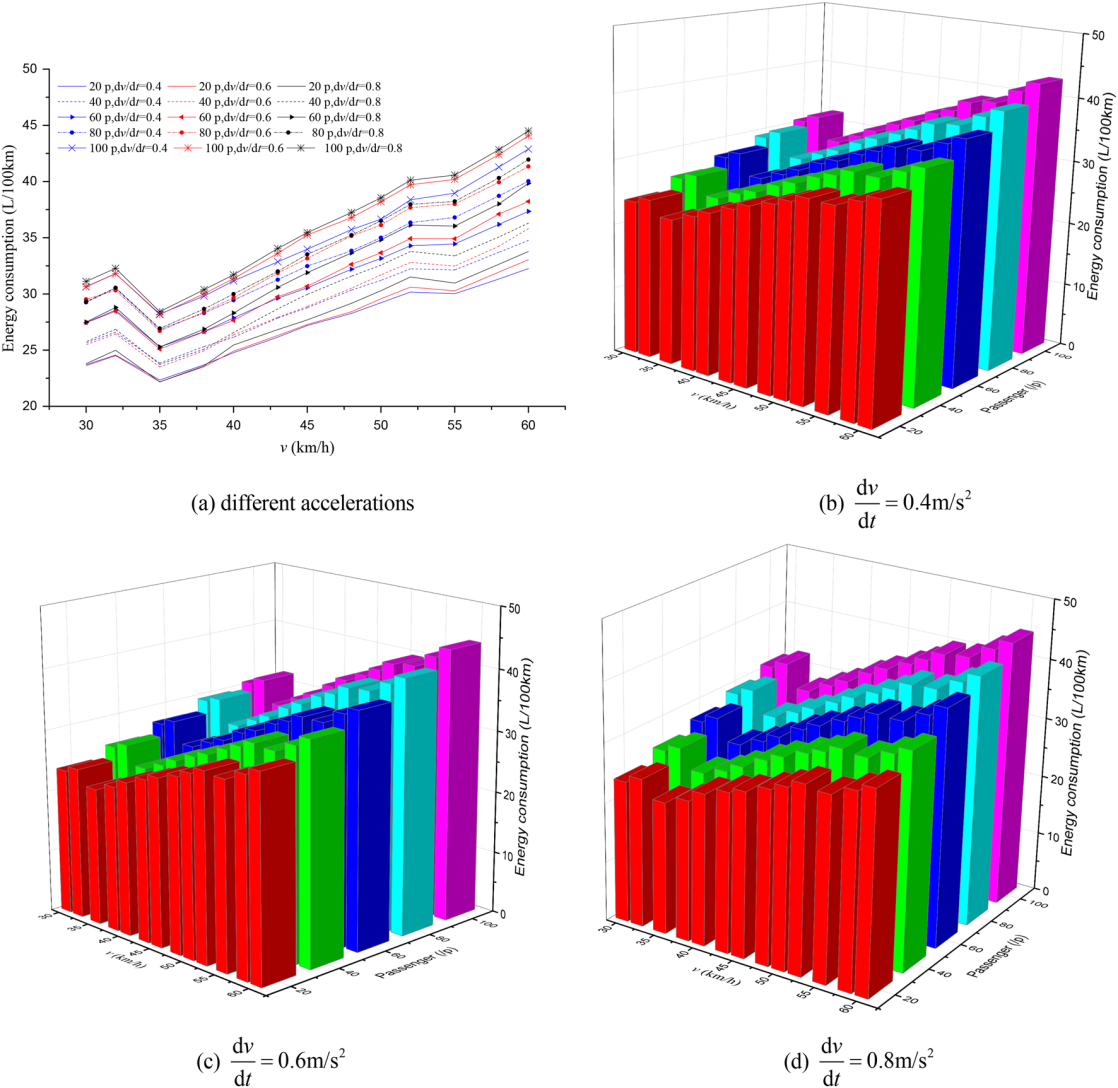
The EC of different passenger load, acceleration and maximum speed for 800 m station spacing.

**Figure 10:**
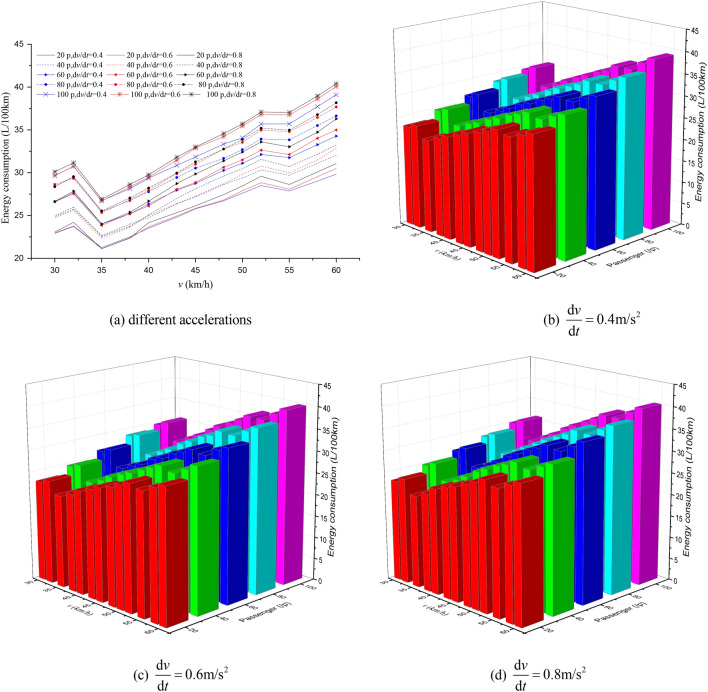
The EC of different passenger load, acceleration and maximum speed for 1000 m station spacing.

**Figure 11:**
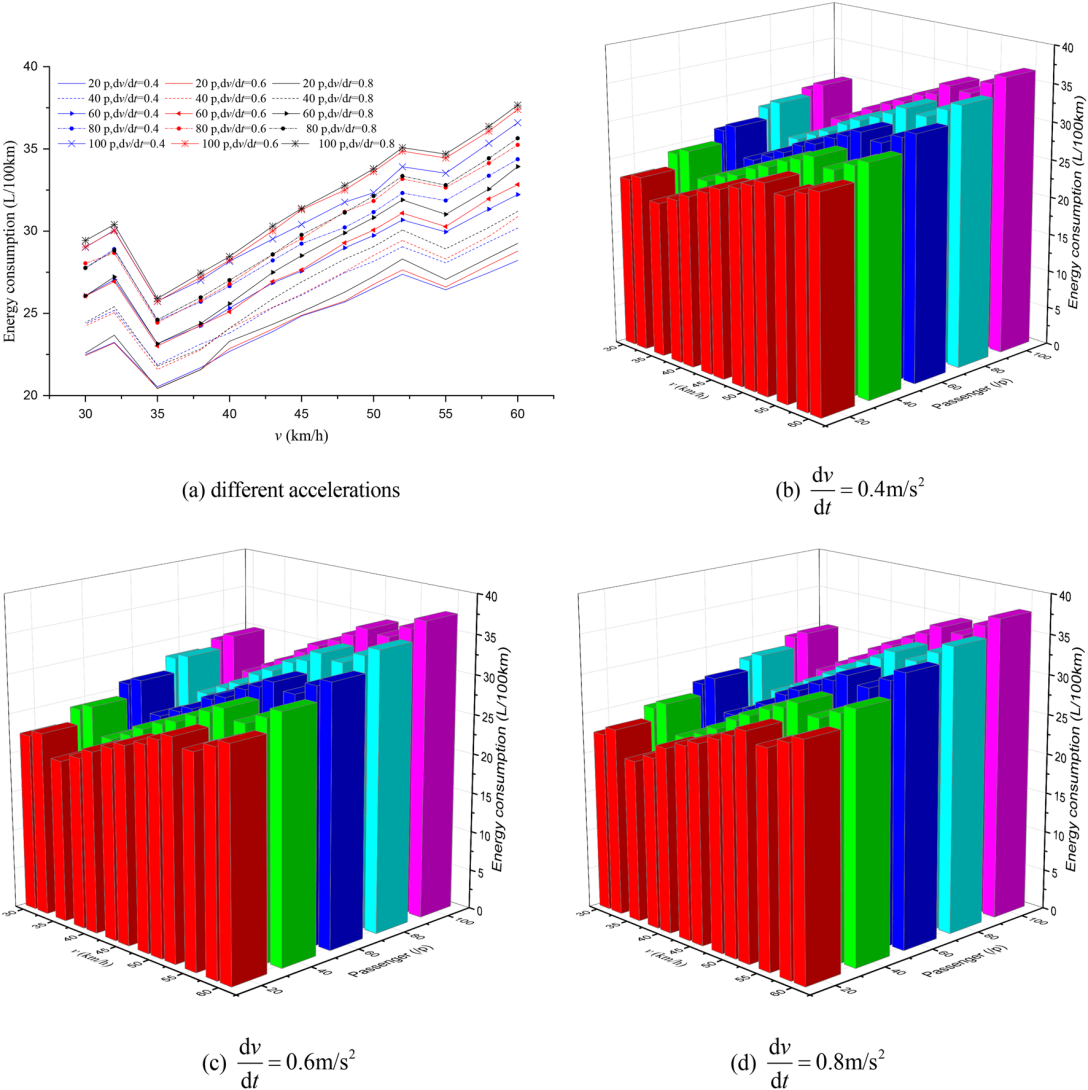
The EC of different passenger load, acceleration and maximum speed for 1200 m station spacing.

**Figure 12:**
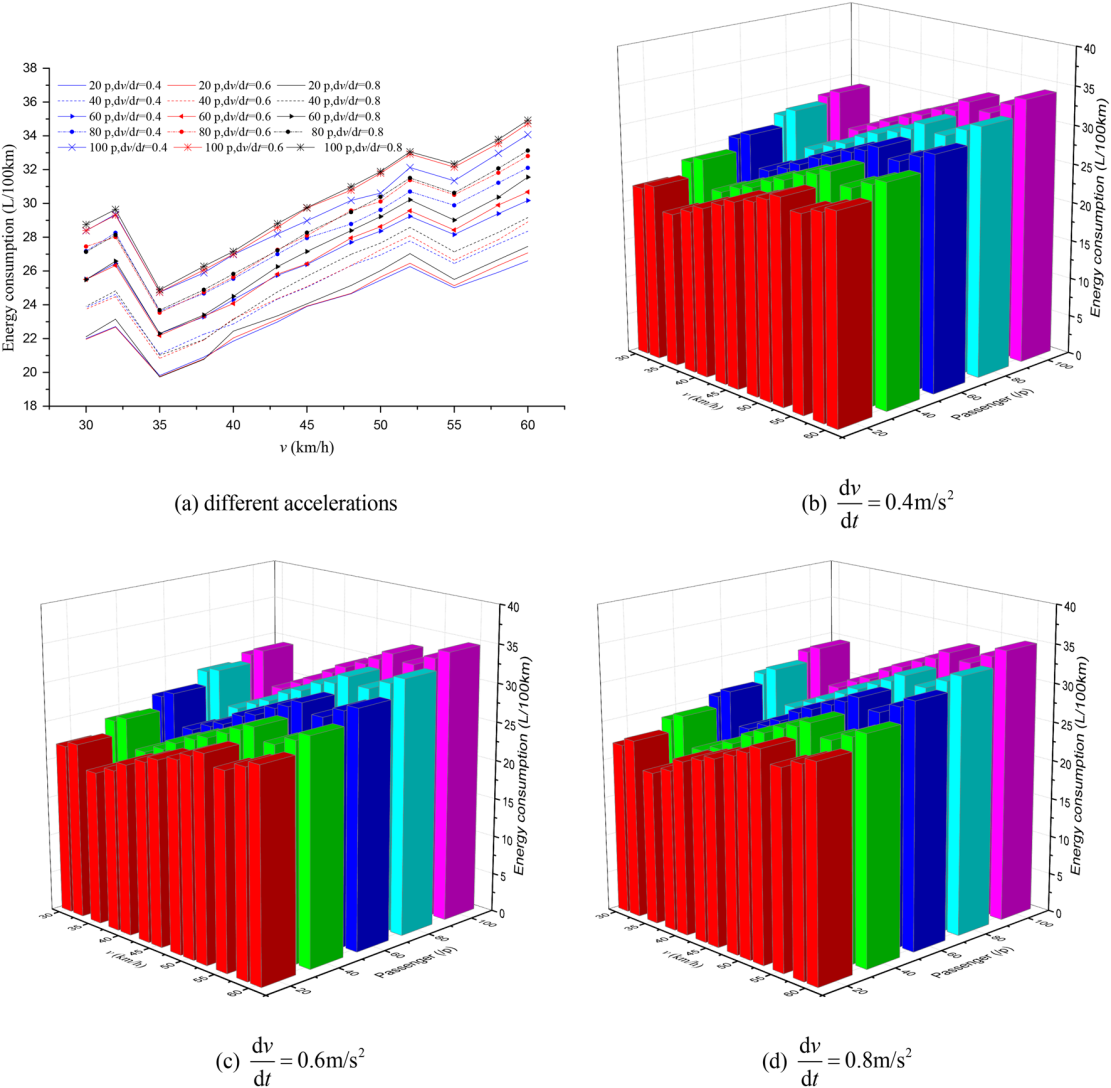
The EC of different passenger load, acceleration and maximum speed for 1500 m station spacing.

Nowadays, Automatic Passenger Counting (APC) systems, such as Wi-Fi-Based APC^42^, infrared APC system^43^, have been usually adopted among public transport companies to collect passenger volume data. Therefore, the number of passengers on the bus can be easily obtained by using APC systems. In this paper, the passenger load values were set at 20, 40, 60, 80 and 100 people (assumed per passenger weight of 65 kg). The impact of passenger load, acceleration, station spacing and maximum speed on EC was assessed based on the simulation framework we designed, which are shown in Figs. 7, 8, 9, 10, 11, 12.

It can be seen from Figs. 7, 8, 9, 10, 11, 12 that the passenger load has a significant impact on EC of buses, and the larger the average station spacing, the lower the EC per 100 km for the same acceleration, maximum speed and passenger load. Generally, the higher the maximum driving speed, the higher the EC of the bus, and the maximum speed with the lowest EC per 100 km is about 35 km/h. For the 400 m station spacing and the $0.4\;{\text{m/s}}^{{2}}$ vehicle acceleration, the maximum speed that the bus can achieve is 50 km/h.

For the lower passenger load (20 ~ 40p), there is little difference in the EC between the acceleration strategies of $0.4\;{\text{m/s}}^{{2}}$,$0.6\;{\text{m/s}}^{{2}}$. For the higher passenger load (60 ~ 100p), the difference of the EC of the three starting strategies increases gradually. The EC of the acceleration strategies of $0.6\;{\text{m/s}}^{{2}}$, $0.8\;{\text{m/s}}^{{2}}$ increases significantly with the increase of maximum speed. In this case, the lower EC of 100 km is the acceleration strategy of $0.4\;{\text{m/s}}^{{2}}$, and the higher EC is the acceleration strategy of $0.8\;{\text{m/s}}^{{2}}$. Thus, acceleration strategies and maximum speed limits are critical factors determining the EC of bus for a certain passenger load and station spacing.

**Figure 13:**
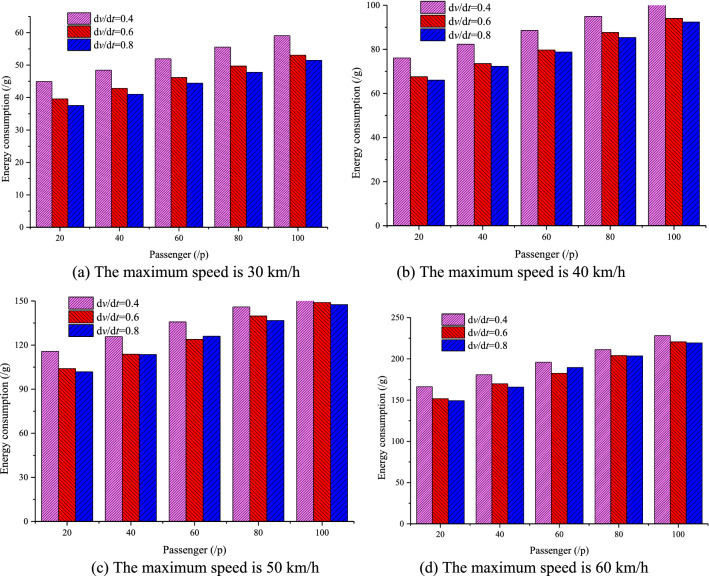
The analysis of EC in acceleration phase.

The EC of different passenger load, acceleration and maximum speed in the acceleration phase is shown in Fig. 13. The results show that with the increase in passenger load, the EC of each acceleration strategy in the acceleration phase is increasing. Moreover, the higher the acceleration, the less EC to reach the maximum speed and the smaller acceleration distance in the acceleration phase.

**Figure 14:**
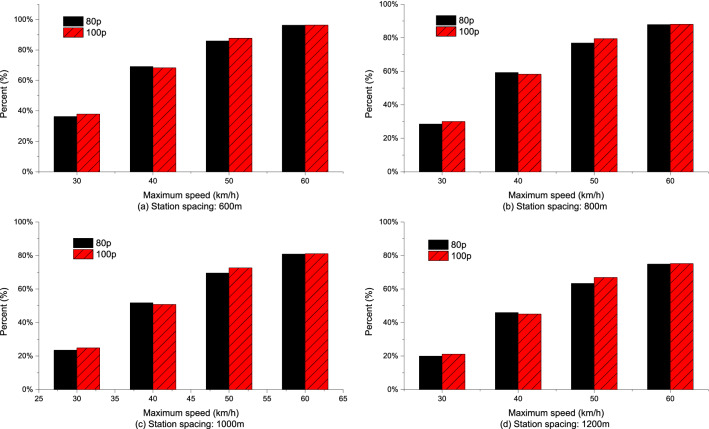
Analysis of the contribution percentage of EC increase in the acceleration phase at $\frac{{{\text{d}}v}}{{{\text{d}}t}} = 0.4\;{\text{m/s}}^{{2}}$.

**Figure 15:**
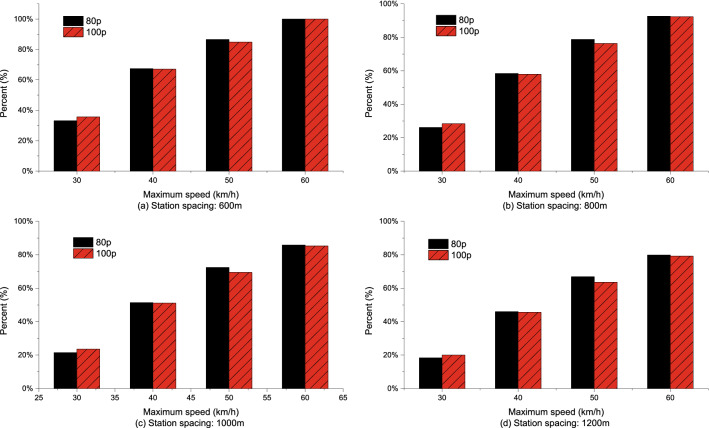
Analysis of the contribution percentage of EC increase in the acceleration phase at $\frac{{{\text{d}}v}}{{{\text{d}}t}} = 0.6\;{\text{m/s}}^{{2}}$.

**Figure 16:**
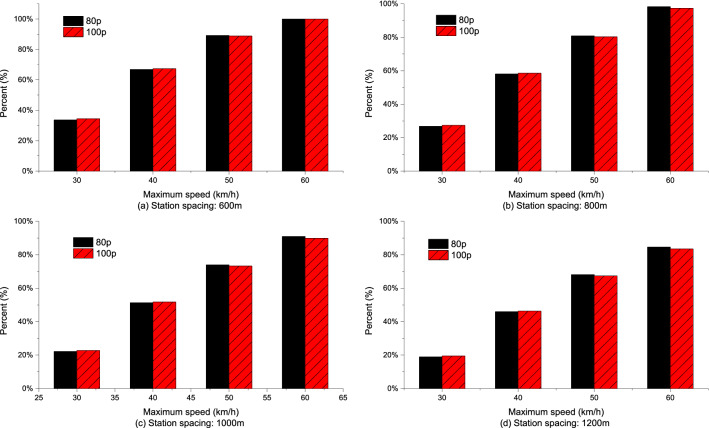
Analysis of the contribution percentage of EC increase in the acceleration phase at $\frac{{{\text{d}}v}}{{{\text{d}}t}} = 0.8\;{\text{m/s}}^{{2}}$.

Compared with the EC of empty buses, the percentages of EC increase in total EC increase in acceleration phase under different station spacing and acceleration strategies are shown in Figs. 14, 15, 16. The analyses showed that for the same station spacing, the acceleration phase is under a greater contribution to the increase of EC. Moreover, the greater the maximum speed limit, the greater the contribution percentage of EC increase in the acceleration phase. For maximum speed of 50–60 km/h, more than 70% of the increase in EC comes from the acceleration phase. In addition, the greater the acceleration, the greater the contribution percentage of EC increase in the acceleration phase. The larger the station spacing, the lower the contribution percentage of energy consumption increase in the acceleration phase.

**Figure 17:**
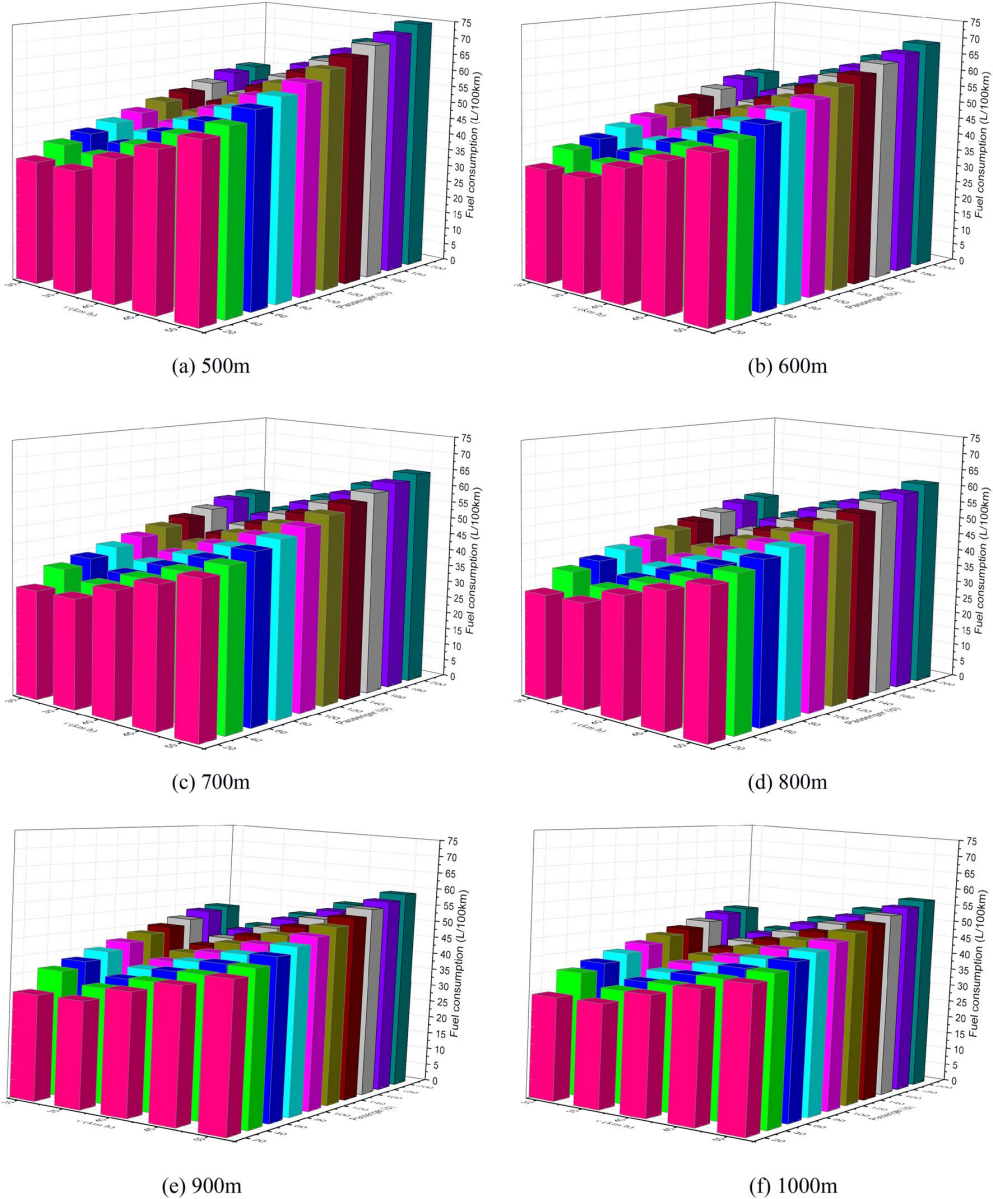
The EC of different passenger load and maximum speed for different station spacings.

Similar results are obtained on the bus with engine WP10.375. Figure 17 shows the impact of passenger load, station spacing and maximum speed on EC of 18-m bus with engine WP10.375 when acceleration is $0.8\;{\text{m/s}}^{{2}}$.

## Conclusions

Reducing energy consumption and promoting sustainable mobility solutions are increasingly becoming key objectives for policymakers worldwide. From this perspective, analyzing of the EC in bus operation is important for creating environmentally friendly cities. In order to identify the factors, such as passenger load, speed and acceleration, that affect significantly EC in bus operation, a simulation framework for describing the level of energy used in different vehicles is provided based on the VEC model which mainly includes the vehicle model, engine model and driver strategy. Furthermore, the correlations between EC, passenger load, vehicle speed and acceleration are analyzed in different station spacing.

The Fonton AUV BJ6123C7MJB with engine WP7.270 is selected for specific analysis of EC by using the simulation framework. The results show that the passenger load has a significant impact on EC of buses, and the larger the average station spacing, the lower the EC per 100 km for the same acceleration, maximum speed and passenger load. Generally, the higher the maximum driving speed, the higher the EC of the bus. Acceleration strategies and maximum speed limits are critical factors determining the EC of bus for a certain passenger load and station spacing. With the increase in passenger load, the EC of each acceleration strategy in the acceleration phase is increasing. Moreover, the higher the acceleration, the less EC to reach the maximum speed and the smaller acceleration distance in the acceleration phase. The analyses showed that for the same station spacing, the acceleration phase is under a greater contribution to the increase of EC. Moreover, the greater the maximum speed limit or the acceleration, the greater the contribution percentage of EC increase in the acceleration phase.

The simulation framework based on VEC model is useful for characterizing bus fuel consumption. This approach should be applied to more types of buses and further evaluated in combination with the actual traffic conditions. Furthermore, the simulation framework will be combined with the dispatching model to study the research of bus energy-saving dispatching. In addition, this simulation framework will also be used to improve the emission of urban public transport.

## Data Availability

The raw data used to support the findings of this study are included within the article.

## References

[1] Huan N, Yao E, Fan Y (2019). Evaluating the environmental impact of bus signal priority at intersections under hybrid energy consumption conditions. Energies.

[2] Agency IE. *Key World Energy Statistics 2019*. 2019; OECD Publishing, Paris

[3] He Y, Rios J, Chowdhury M (2012). Forward power-train energy management modeling for assessing benefits of integrating predictive traffic data into plug-in-hybrid electric vehicles. Transp. Res. Part D Transp. Environ..

[4] Xia H (2014). Eco-approach and departure techniques for connected vehicles at signalized traffic intersections.

[5] Fontaras G, Zacharof N, Ciuffo B (2017). Fuel consumption and CO2 emissions from passenger cars in Europe-Laboratory versus real-world emissions. Prog. Energy Combust. Sci..

[6] Wang J, Rakha H (2016). Fuel consumption model for conventional diesel buses. Appl. Energy..

[7] Merkisz J, Rymaniak Ł (2017). Tests of urban bus specific emissions in terms of currently applicable heavy vehicles operating emission regulations. Combust. Eng..

[8] e Silva JDA, Moura F, Garcia B, Vargas R (2015). Influential vectors in fuel consumption by an urban bus operator: Bus route, driver behavior or vehicle type?. Transp. Res. Part D Transp. Environ..

[9] Frey H, Rouphail N, Zhai H (2007). Comparing real-world fuel consumption for diesel- and hydrogen-fueled transit buses and implication for emissions. Transp. Res. Part D Transp. Environ..

[10] Wang A, Ge Y, Tan J (2011). On-road pollutant emission and fuel consumption characteristics of buses in Beijing. J. Environ. Sci..

[11] Zhang S, Wu Y, Liu H (2014). Real-world fuel consumption and CO2 emissions of urban public buses in Beijing. Appl. Energy..

[12] Ma H, Xie H, Huang D (2015). Effects of driving style on the fuel consumption of city buses under different road conditions and vehicle masses. Transp. Res. Part D Transp. Environ..

[13] Mišanović M, Živanović M, Tica S (2015). Energy efficiency of different bus subsystems in Belgrade public transport. Thermal Sci..

[14] Ahn K, Rakha H, Trani A (2002). Estimating vehicle fuel consumption and emissions based on instantaneous speed and acceleration levels. J. Transp. Eng..

[15] Cai T, Wang X, Chen C (2009). Modeling and verification of fuel consumption calculating model for passenger vehicles. J. Highw. Transp. Res. Dev..

[16] Peng B, Du H, Ma S (2014). Road vehicle energy consumption and emissions based on LEAP model. J. Wuhan Univ. Technol..

[17] Tang X, Zhang X, Sun H (2012). Energy consumption models for urban bus transport. J. Transp. Syst. Eng. Inf. Technol..

[18] Okafor I, Unachukwu G, Odukwe A (2014). Measuring energy efficiency of the public passenger road transport vehicles in Nigeria. Transp. Policy..

[19] Masikos M, Demestichas K, Adamopoulou E (2014). Mesoscopic forecasting of vehicular consumption using neural networks. Soft. Comput..

[20] Ivković Ivan S, Kaplanović Snežana M, Milovanović BM (2017). Influence of road and traffic conditions on fuel consumption and fuel cost for different bus technologies. Therm. Sci..

[21] Song G, Yu L (2010). Distribution characteristics and models of Vehicle Specific Power on urban expressways. J. Transp. Syst. Eng. Inf. Technol..

[22] Wu Y, Yu L, Song G (2013). Feasibility study of fuel consumption prediction model by integrating vehicle-specific power and controller area network bus technology. Transp. Res. Rec..

[23] Holmén B, Sentoff K (2015). Hybrid-Electric passenger car carbon dioxide and fuel consumption benefits based on real-world driving. Environ. Sci. Technol..

[24] Duarte G, Gonçalves G, Baptista P (2015). Establishing bonds between vehicle certification data and real-world vehicle fuel consumption –a Vehicle Specific Power approach. Energy Convers. Manage..

[25] Wang J, Rakha H (2017). Convex fuel consumption model for diesel and hybrid buses. Transp. Res. Rec..

[26] Xu Z, Wei T, Easa S (2018). Modeling relationship between truck fuel consumption and driving behavior using data from internet of vehicles. Comput. Aided Civ. Inf. Eng..

[27] Yu Q, Li T, Li H (2016). Improving urban bus emission and fuel consumption modeling by incorporating passenger load factor for real world driving. Appl. Energy..

[28] Chen X, Shan X, Ye J (2017). Evaluating the effects of traffic congestion and passenger load on feeder bus fuel and emissions compared with passenger car. Transp. Res. Procedia..

[29] Fredy D, Natalia F, José-María L (2021). Effects of passenger load, road grade, and congestion level on real-world fuel consumption and emissions from compressed natural gas and diesel urban buses. Appl. Energy.

[30] Ehsani M, Ahmadi A, Fadai D (2016). Modeling of vehicle fuel consumption and carbon dioxide emission in road transport. Renewab. Sustainable Energy Rev..

[31] He R, Zhuang Z, Zheng J (2007). Interval mathematics analysis method of running parameters influence on automobile fuel economy. J. Traffic Transp. Eng..

[32] Bao F (2011). Research on calculation method of fuel consumption rate base on engine universal characteristics. Adv. Mater. Res..

[33] Chaim M, Shmerling E (2013). A model for vehicle fuel consumption estimation at urban operating conditions. Int. J. Mech..

[34] Schwickart T, Voos H, Hadji-Minaglou J (2015). Design and simulation of a real-time implementable energy-efficient model-predictive cruise controller for electric vehicles. J. Franklin Inst..

[35] Yang X, Liu L (2020). A multi-objective bus rapid transit energy saving dispatching optimization considering multiple types of vehicles. IEEE Access..

[36] Handbook C (2011). Fundamentals, Driving Dynamics, Components, Mechatronics, Perspectives.

[37] Wang Q, Huo H, He K (2008). Characterization of vehicle driving patterns and development of driving cycles in Chinese cities. Transp. Res. Part. D Transp. Environ..

[38] Mahesh S, Ramadurai G (2017). Analysis of driving characteristics and estimation of pollutant emissions from intra-city buses. Transp. Res. Procedia..

[39] Zener O, Zkan M (2020). Fuel consumption and emission evaluation of a rapid bus transport system at different operating conditions. Fuel.

[40] Zheng B, Huang X, Zhang W (2018). Adhesion characteristics of tire-asphalt pavement interface based on a proposed tire hydroplaning model. Adv. Mater. Sci. Eng..

[41] Zu Y, Wang B, Gu J (2018). Explanation of national standard guideline on the design of traffic operation of urban roads. Transp Res..

[42] Nitti M, Pinna F, Pintor L (2020). iABACUS: A Wi-Fi-Based automatic bus passenger counting system. Energies.

[43] Olivo A, Maternini G, Barabino B (2019). Empirical study on the accuracy and precision of automatic passenger counting in European bus services. Open Transp. J..

